# Optimization of the Encapsulation of Vitamin D3 in Oil in Water Nanoemulsions: Preliminary Application in a Functional Meat Model System

**DOI:** 10.3390/foods13172842

**Published:** 2024-09-07

**Authors:** Nallely Peñuñuri-Pacheco, Yuvitza Alejandra Moreno-García, Humberto González-Ríos, Humberto Astiazarán-García, Yolanda L. López-Franco, Orlando Tortoledo-Ortiz, Anna Judith Pérez-Báez, José Luis Dávila-Ramírez, Jaime Lizardi-Mendoza, Martin Valenzuela-Melendres

**Affiliations:** 1 Coordinación de Tecnología de Alimentos de Origen Animal, Centro de Investigación en Alimentación y Desarrollo, Carretera Gustavo Enrique Astiazarán Rosas No. 46, La Victoria, Hermosillo 83304, Sonora, Mexico; npenunuri222@estudiantes.ciad.mx (N.P.-P.); ymoreno122@estudiantes.ciad.mx (Y.A.M.-G.); hugory@ciad.mx (H.G.-R.); lopezf@ciad.mx (Y.L.L.-F.); jose.davila@ciad.mx (J.L.D.-R.); jalim@ciad.mx (J.L.-M.); 2 Departamento de Ciencias Químico-Biológicas, Universidad de Sonora, Hermosillo 83000, Sonora, Mexico; hastiazaran@ciad.mx; 3 Coordinación de Nutrición, Centro de Investigación en Alimentación y Desarrollo, Carretera Gustavo Enrique Astiazarán Rosas No. 46, La Victoria, Hermosillo 83304, Sonora, Mexico; otortoledo@ciad.mx; 4 Instituto de Acuacultura del Estado de Sonora, Comonfort y Paseo del Canal, Centro de Gobierno, Ed. Sonora, Hermosillo 83280, Sonora, Mexico; anna.perez@sonora.gob.mx

**Keywords:** functional meat product, vitamin D3, encapsulation

## Abstract

Meat products containing Vitamin D3 (VD3) are an innovative option that could contribute to reducing deficiencies in this micronutrient. Designing nanoemulsions that carry VD3 is the first step in developing functional meat products. Thereby, this study investigated the impact of food components on the nanoemulsion properties. A central composite design was used to study the effects of pea protein (PP, 0.5–2.5%), safflower oil (SO, 5–15%), and salt (0–0.5%) on the nanoemulsion stability (ζ-potential and particle size) and the VD3 retention. Also, the optimized nanoemulsion carrying VD3 was incorporated into a meat matrix to study its retention after cooking. The combination of food components in the optimized nanoemulsion were SO = 9.12%, PP = 1.54%, and salt content = 0.4%, resulting in the predicted values of ζ-potential, particle size, and VD3 retention of −37.76 mV, 485 nm, and 55.1%, respectively. The VD3 that was nanoencapsulated and included in a meat product remained more stable after cooking than the VD3 that was not encapsulated. If a meat product is formulated with 5 or 10% safflower oil, the stability of the nanoencapsulated VD3 is reduced. This research contributes to developing functional meat products carrying nanoencapsulated vitamin D3 in natural food-grade components.

## 1. Introduction

Vitamin D is a micronutrient that plays a crucial role in our body. It helps with calcium absorption and bone and mineral metabolism. It regulates intestinal absorption, renal excretion of calcium and phosphorus, and bone resorption [1]. However, several factors can lead to a vitamin D deficiency, such as insufficient sun exposure, an unbalanced diet, full-cover clothing, and the use of sunscreen [2,3]. This deficiency is a global health issue affecting over a billion people [4]. In Mexico, vitamin D deficiency affects 27.3% of preschool children and 17.2% of school children [5]. Women of reproductive age and older adults also suffer from this condition, with a prevalence of 31.6% and 63.7%, respectively [6,7].

Foods that are naturally rich in vitamin D are scarce; some of the most well-known sources include fatty fish, eggs, and mushrooms exposed to UV light [8]. However, these food sources may not be readily available or may be too expensive for some people. Therefore, fortifying foods with vitamin D is an excellent strategy to increase food options and reduce vitamin D deficiency among the population [9]. Foods fortified with vitamin D are limited, with the best-known sources being milk, cereals, and juices. Expanding the availability of vitamin D-enriched foods is necessary to prevent deficiencies among the population [10,11]. Meat products are a great source of essential nutrients like a high protein content, vitamin B complex, iron, zinc, and selenium [12], making them a good option for fortification with vitamin D. However, for some people, meat products are considered unhealthy due to their high content of saturated fatty acids, cholesterol, and salt [13]. These ingredients are linked to cardiovascular diseases and certain types of cancer [14,15]. A healthy alternative for the sector of the population who are concerned about their health is in the field of functional foods.

In a functional meat product, the animal fat can be replaced with vegetable oils to reduce the saturated fatty acids (SFA), increase the polyunsaturated fatty acids (PUFA), and reduce the cholesterol content [16]. Additionally, the salt content can be reduced [17], or vegetable proteins in the formulation can be used to increase the nutritional profile [18]. Using safflower oil is a good option for developing a functional meat product, as evidenced by Kang et al. [19] and Kaynakci and Kilic [20]. Also, according to research conducted by Shen et al. [21] and Broucke et al. [22], another option for creating functional foods is to use pea protein in the meat product formulation. 

Meat products that have been reformulated by changing the fatty acid profile, reducing the cholesterol content, decreasing salt, and increasing the protein content could be the healthiest options to be used as vitamin D carrier systems, resulting in increased attractiveness to a larger group of consumers who are concerned about their health [23]. Research in this field is limited. Gomes et al. [24] conducted a study on the potential application of a mixture of olive and linseed oils as a backfat replacer and vitamin D as a supplement to develop a beef patty enriched with polyunsaturated fatty acids and vitamin D. The authors highlight that the appropriate combination of the ingredients used in the newly developed formulation could be used to develop new and attractive fresh meat products with health properties. 

This study underlines the need for research on functional meat products as a potential carrier system for vitamin D. However, the fortification of such foods with vitamin D can be challenging due to factors like sensitivity to high temperature and light, exposure to oxygen, and interactions with other components in the food matrix. These factors can impact the stability and absorption of vitamin D when the food is consumed [25]. The loss of vitamin D from fortified foods could be reduced by using nanoemulsions [26]. However, it is essential to note that the current research on nanoemulsions as carriers for vitamin D primarily focuses on the use of artificial components such as tween-20 and Sunsoft A-12 (decaglycerol monolaurate) [27], tween-80 [28], Kolliphor^®^ HS 15 or Kolliphor^®^ RH-40 (hydrophilic surfactant derivatives of polyethylene glycol) [25,29], and Pluronic^®^ F-127 (non-ionic copolymer surfactant) [30], among others. This highlights the urgent need to increase our knowledge about food-grade nanoemulsion carriers of vitamin D with natural components and their potential incorporation into functional meat products. 

To develop food-grade nanoemulsions with natural components, it is possible to use the same ingredients found in a functional meat product, such as safflower oil and pea protein [20,21]. In addition to enhancing the nutritional and functional value of new meat products, these ingredients could be used to develop vitamin D-carrying nanoemulsions, simplifying the formulation of new meat products [31,32]. However, the stability and ability to retain vitamin D in these nanoemulsions could be affected by several factors, including the concentration of pea protein [33], the oil content [31], and the salt concentration in the system. Salt is essential in meat products as it helps to solubilize myofibrillar proteins [34]. When used in nanoemulsion preparation with plant proteins, salt has a significant impact on the stability and encapsulation capacity of the bioactive components [35]. 

Since multiple factors influence the nanoemulsion stability and the encapsulation efficiency of the bioactive compounds, it is essential to optimize the component proportions in the system before incorporating them into the food. The response surface methodology (RSM) is an appropriate statistical methodology to study the effects and interactions of the independent variables on the response variables to aid in optimizing the system’s component proportions. Researchers have used this technique to optimize the preparation of vitamin D nanoemulsions [25,28,36]. Therefore, as a first step, this research aimed to study the effects and interactions of the pea protein, safflower oil, and salt on the nanoemulsions’ stability and vitamin D retention capacity and assist in their optimization using the response surface methodology. In the second step, the optimized nanoemulsion carrying the vitamin D3 was integrated into a meat batter with varying percentages of safflower oil incorporated (simulating a functional meat product), and the vitamin D stability after cooking was assessed.

## 2. Materials and Methods

### 2.1. Materials and Reagents

Ground beef (semimembranosus, 48 h postmortem, pH 5.9, 95% lean, 5% fat) was obtained from the local supermarket, pea protein isolate (80% protein) was obtained from Ingredion^®^ (Jalisco, Mexico), vitamin D3 was obtained from Sigma-Aldrich (St. Louis, MO, USA), safflower oil (Oleico^®^, Walmart, Hermosilo, Mexico) and salt (Salt Bay^®^, Waltmart, Hermosillo, Mexico) were purchased from a local supermarket. Deionized water was used to prepare the nanoemulsions and solutions.

### 2.2. Experimental Design

In the first experiment, the effects and interactions of the independent variables including the safflower oil (5.0, 7.03, 10.0, 12.97, and 15%), pea protein (0.5, 0.91, 1.5, 2.09, and 2.5%), and salt (0.0, 0.1, 0.25, 0.4, and 0.5%) on the response variables such as the ζ-potential, particle size, and vitamin D3 retention were evaluated using a central composite design (CCD). The CCD included eight factorial points, six axial points, and six central points for a total of twenty experimental runs. All experiments were replicated twice, and each was performed in triplicate. The experimental runs and the response variables evaluated are summarized in Table 1. The optimal combination of the nanoemulsion components (safflower oil, pea protein, and salt) to minimize the ζ-potential and particle size of the nanoemulsion and maximize the vitamin D3 retention was estimated by response surface methodology. 

In the second experiment, the optimized nanoemulsion obtained in the first part of the study was incorporated into a functional meat model system as an exploratory study on the stability of vitamin D3 in a more complex food matrix. A 3 × 2 factorial arrangement was used to determine the effects of including the safflower (0, 5, and 10%) and vitamin D3 condition (encapsulated in a nanoemulsion or unencapsulated, called free) in a meat batter on the vitamin D3 retention after cooking. Each treatment combination was carried out in triplicate. 

### 2.3. Nanoemulsion Preparation

Nanoemulsions were prepared by mixing the safflower oil containing vitamin D3 (130 µg/g oil) as the disperse phase with the deionized water containing pea protein and salt as the continuous phase. The proportions of the components used in the nanoemulsion preparation are detailed in Table 1. A coarse emulsion was created by homogenizing the components at 18,800 rpm for 4 min, using a high-speed mixer (Ultraturrax^®^ T25 basic homogenizer from IKA Works in Wilmington, NC, USA). The coarse emulsion was further homogenized using an ultrasonic homogenizer (JY 92-IIDN, Stratford, CT, USA) at 20 kHz for 8 min while maintaining the temperature between 0 and 2 °C.

### 2.4. ζ-Potential and Particle Size

The ζ-potential measures the electric charge at the surface of a particle, providing crucial information about the repulsive forces between nanoparticles. Increasing the electrostatic repulsion between the droplets and reducing the particle size helps prevent coalescence and flocculation, resulting in a more stable emulsion. Therefore, it is essential to measure the ζ-potential, particle size, and vitamin D3 retention when optimizing nanoemulsions [29]. The ζ-potential and particle size of the nanoemulsion was determined using a dynamic light scattering analysis with a Möbiuz instrument (Wyatt Technology Corp, Santa Barbara, CA, USA) equipped with a laser at 532 nm and a scattering angle of θ = 163.5°. The analysis was conducted in three replicates, each lasting for 30 s at a temperature of 2 °C. The average values of the replicates were reported.

### 2.5. Determination of Vitamin D3

The vitamin D3 retention in the nanoemulsions and meat samples was determined by HPLC. Three grams of the nanoemulsions or meat samples containing vitamin D3 were vortexed with 30 mL of chloroform–methanol (2:1) at 25 °C for 3 min. Subsequently, the solution was filtered through Whatman No 1 filter paper and dried under nitrogen flow. The obtained extract was diluted with chloroform–methanol in a 1:3 (*v*/*v*) ratio, filtered with a 0.45 µm PTFE filter (Pall Corporation, East Hills, NY, USA), and analyzed by HPLC. A high-performance liquid chromatography instrument (Agilent Technologies 1220 infinity, Palo Alto, CA, USA) equipped with a diode array detector was used to quantify the Vitamin D3. Chromatographic separation was performed using a Hypersil ODS 2 C18 column (5 μm, 250 × 4.6 mm, Thermo Scientific, Waltham, MA, USA). The mobile phase was pure methanol (100%) at a 1 mL/min flow rate at 25 °C. Detection was carried out at a wavelength of 265 nm. For the quantification of vitamin D3, the calibration curve included the range of concentrations used, from 2 to 200 μg (r^2^ = 0.995). 

The vitamin D3 retention in the nanoemulsions and meat samples was calculated using the following Equation (1):

(1) $$Vitamin\;D3\;retention\;\left(\%\right)=\frac{V_{D3-A}}{V_{D3-B}}\times 100$$

where VD3-A is the vitamin D3 concentration in the nanoemulsion or meat sample, and VD3-B is the vitamin D3 initially incorporated during the nanoemulsion or meat sample preparation. 

### 2.6. Stability of Vitamin D3 in a Functional Meat Model System

Beef meat was trimmed of visible fat and connective tissue and ground through a 5 mm plate (Hobart grinder, model 4152, Troy, OH, USA). Ground meat (95% lean, 5% fat) was tempered at 2 °C for one hour and used immediately. Ground meat was washed twice in a phosphate buffer (50 mM, pH 7) at 2 °C for 10 min. After discarding the liquid by decanting, the meat was combined with 1 volume of saline solution (NaCl 0.5 M), homogenized at 8000 rpm for 3 min (Ultraturrax^®^ T25 basic homogenizer from IKA Works in Wilmington, NC, USA), and allowed to rest overnight at 2 °C. The resulting meat batter was used to prepare a functional meat model system by incorporating safflower oil (SO at 0, 5 and 10%) and vitamin D3 (VD3), encapsulated in a nanoemulsion (nE) or unencapsulated (free). The nanoemulsion used in this part of the study corresponds to the one obtained from the optimization process, as described in Section 2.2. The resulting combinations of treatments were as follows: (1) 0% SO + VD3 nE, (2) 0% SO + VD3 free, (3) 5% SO + VD3 nE, (4) 5% SO + VD3 free, (5) 10% SO + VD3 nE, and (6) 10% SO + VD3 free. All treatments were incorporated with 7 µg/g of VD3 (encapsulated in a nanoemulsion or unencapsulated). The safflower oil was mixed with the meat batter using a high-speed homogenizer at 10,000 rpm for 3 min at 2 °C. Treatments were cooked until they reached a temperature of 71 °C, cooled on ice, and stored at 2 °C. After 24 h of the functional meat model system resting, vitamin D3 retention was measured as described in Section 2.5. The experiment was replicated twice, and the vitamin D3 retention in each treatment was determined in triplicate.

### 2.7. Statistical Analysis 

The experimental data were adjusted to a second-order polynomial model in the first part of the study. The general model corresponding to the central composite design is as follows (Equation (2)):

(2) $$y=\beta_{0}+\sum_{i=1}^{3}\beta_{i}X_{1}+\sum_{i=1}^{3}\beta_{ii}X_{i}^{2}+\sum_{i<j}\beta_{ij}X_{i}X_{j}+\varepsilon$$

where *y* is the response variable (ζ-potential, polydispersity index, particle size, and vitamin D3 retention); *β*_0_, *β_i_*, *β_ii_*, and *β_ij_* are the intercept and the linear, quadratic, and interaction coefficients, respectively; and *X_i-j_* are the independent variables (safflower oil, pea protein, and salt). The analysis of variance (ANOVA), coefficient of determination (R^2^), and lack of fit were determined to evaluate the adequacy of the models using Design Expert (V.13.0.5.0, Stat-Ease, Inc., Minneapolis, MN, USA). The statistical significance of the model and model coefficients was determined at a probability level of *p* < 0.05. The graphical optimization procedures were carried out to predict the optimal combination of independent variables to obtain a minimum value of ζ-potential, particle size, and maximum value of vitamin D3 retention. A nanoemulsion resulting from the optimal combination of independent variables was experimentally prepared for model validation, and the response variables were measured as described in the previous sections. The experimental and predicted response variables were statistically compared. 

In the second part of the study, data were processed using the Number Cruncher Statistical Systems 2023 (NCSS, Kaysville, UT, USA) software. Two-way analysis of variance (ANOVA) was used to evaluate the differences among treatments (*p* < 0.05), where the safflower oil (0, 5, and 10%) and vitamin D3 condition (encapsulated in a nanoemulsion or unencapsulated, called free) were assigned as fixed effects and the replications as random effects. When significant differences between treatments were found, the Tukey–Kramer test was performed (*p* < 0.05).

## 3. Results and Discussion

### 3.1. ζ-Potential, Particle Size, and Vitamin D3 Retention

Table 1 shows the results for each one of the 20 experimental runs. The values range between −4.85–−43.86 mV, 232–3815 nm, and 25.1–79.9% for ζ-potential, particle size, and vitamin D3 retention, respectively. Table 2 shows the regression coefficients, coefficients of determination (R^2^), and significance of the models for all the response variables studied. 

**Figure 1:**
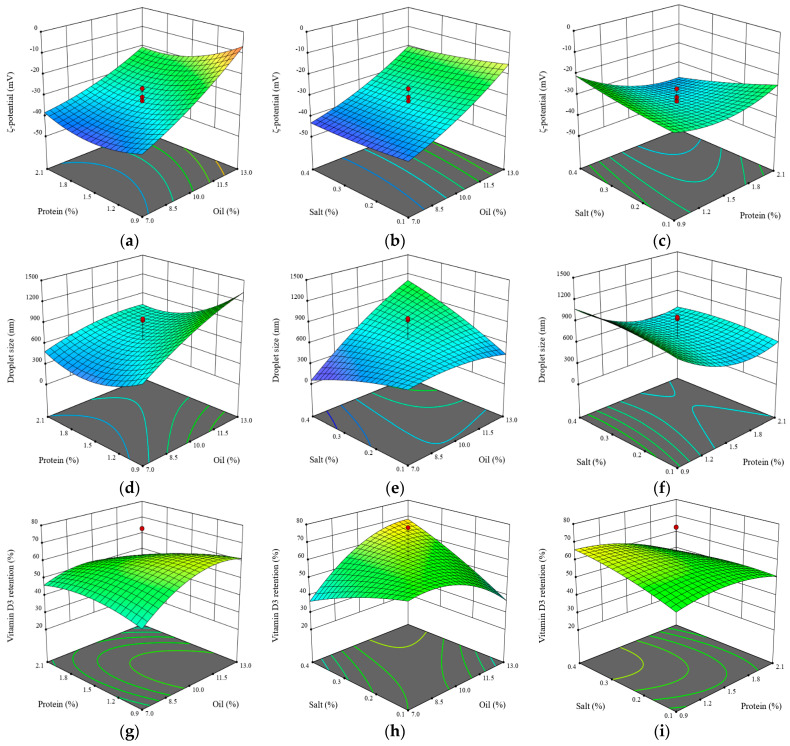
Effect of protein and oil (**a**), salt and oil (**b**), and salt and protein (**c**) on the ζ-potential; protein and oil (**d**), salt and oil (**e**), and salt and protein (**f**) on the droplet size; and protein and oil (**g**), salt and oil (**h**), and salt and protein (**i**) on the vitamin D3 retention.

The models used to estimate the ζ-potential, particle size, and vitamin D3 retention were found to be significant. None of the models exhibited a lack of fit (*p* > 0.05), and the R^2^ values ranged between 0.74 and 0.93. These statistical values indicate that these models were suitable for predicting the response variable as a function of SO, PP, and salt levels in the nanoemulsion formulation. In addition, Figure 1 shows these effects as 3D graphs where the direction of the effects of the independent variables on the ζ-potential (Figure 1a–c), particle size (Figure 1d–f), and vitamin D3 retention (Figure 1g–i) can be seen. According to the models shown in Table 2, the ζ-potential and the particle size were affected (*p* < 0.05) by the linear terms of SO and PP, the interactive term between PP and salt, and the quadratic term of PP. The absolute value of the ζ-potential decreases, and the particle size increases (*p* < 0.05), as the SO is incorporated into the nanoemulsion. In contrast, an increase in the amount of PP had the opposite effect on these response variables. The ζ-potential measures the electric charge at the surface of a particle, providing insight into the repulsive forces between nanoparticles [29]. Increasing the electrostatic repulsive force between the droplets and decreasing the particle size prevents coalescence and flocculation, resulting in a more stable emulsion [37]; therefore, the measurement of the ζ-potential and the particle size are critical markers of the nanoemulsion stability. The ζ-potential was affected by the salt x PP interaction, while the particle size was affected by the salt x SO interactions (*p* < 0.05), as can be seen in Figure 1c,e, respectively, which resulted in a saddle-shaped response. The imbalance between the continuous and disperse phases results in a nanoemulsion with less repulsive force and an increase in particle size, and hence less stability. The results obtained in our study are in agreement with the research conducted by Maurya and Aggarwal [29], who evaluated the impact of oil (caprylic-/capric triglyceride) and surfactant (Kolliphor^®^ HS 15) in a nanoemulsion on the particle size and ζ-potential. The authors reported that an increase in the oil phase from 10 to 25% at a constant surfactant concentration increases the particle size and decreases the ζ-potential. When the authors evaluated the impact of the surfactant concentration, they observed a contrary effect on these response variables. Similar results were reported by Mehmood et al. [28], who studied the effect of the surfactant/oil ratio (soy lecithin and tween 80/canola oil) on the droplet size. When the surfactant/oil ratio increased, the droplet size decreased due to a reduction in the interfacial tension in the system. 

The ζ-potential is also affected (*p* < 0.05) by the interactive term of salt and PP (Table 2). The 3D graph in Figure 1c shows that the absolute value of the ζ-potential decreases as the salt content increases in a nanoemulsion with a low concentration of PP. According to Wang et al. [35], the observed effect could be due to Na^+^ and Cl^−^ ions binding to the –COO− and ^−^NH^3+^ groups of the proteins, respectively. This results in ion binding and electrostatic shielding effects at high salt concentrations. A similar trend was reported by Maurya and Aggarwal [29] in nanoemulsions exposed to salt concentrations in the range of 0 to 750 mM after 30 days of cold storage. The authors reported a decrease in the absolute value of the ζ-potential from −18.59 at 0 mM of NaCl to −9.26 at 750 mM of NaCl.

Table 2 also shows the regression model used to predict vitamin D retention in the nanoemulsion. According to this model, vitamin D3 retention was affected (*p* < 0.05) by the interactive term of SO and salt, and the quadratic term of SO, resulting in a saddle shaped response (Figure 1h). The vitamin D3 retention was lower at higher salt concentrations and gradually increased with a rise in the disperse phase (SO) when the concentration of PP remained constant at around 1.5%. This combination of factors (high levels of salt and SO, and a PP concentration of around 1.5%) seems to have attained the maximum vitamin D3 retention. An emulsifier concentration of around 1.5% decreases the degradation of vitamin D, possibly by creating a rigid surfactant shell at the water-oil interface. This prevents the repulsion of vitamin D and reduces new surface formation, thereby enhancing the stability of the vitamin D [28]. On the other hand, as shown in Figure 1h, increasing the SO content in a system with the highest salt levels (0.5%) leads to an increased retention of vitamin D3, possibly due to the formation of larger droplets (Figure 1e), which results in a lower surface area. Additionally, the hydrophobic nature of vitamin D3 and the tendency of SO to entrap it also contribute to this increase in retention [27]. The results found in our study follow a similar tendency to those reported by Almouazen et al. [38], who reported an increase in the encapsulation efficiency of vitamin D3 as the oil content increased in a nanoemulsion prepared with polylactic acid (polymer) and Miglyol^®^829 (caprylic/capric/succinic triglyceride). Maurya and Aggarwal [29] also reported an increase in the encapsulation efficiency of vitamin D3 when the oil content was increased from 10 to 35% in a nanoemulsion prepared with Monegyl caprylic/capric triglyceride and Kolliphor^®^ HS 15. 

### 3.2. Optimization

**Figure 2:**
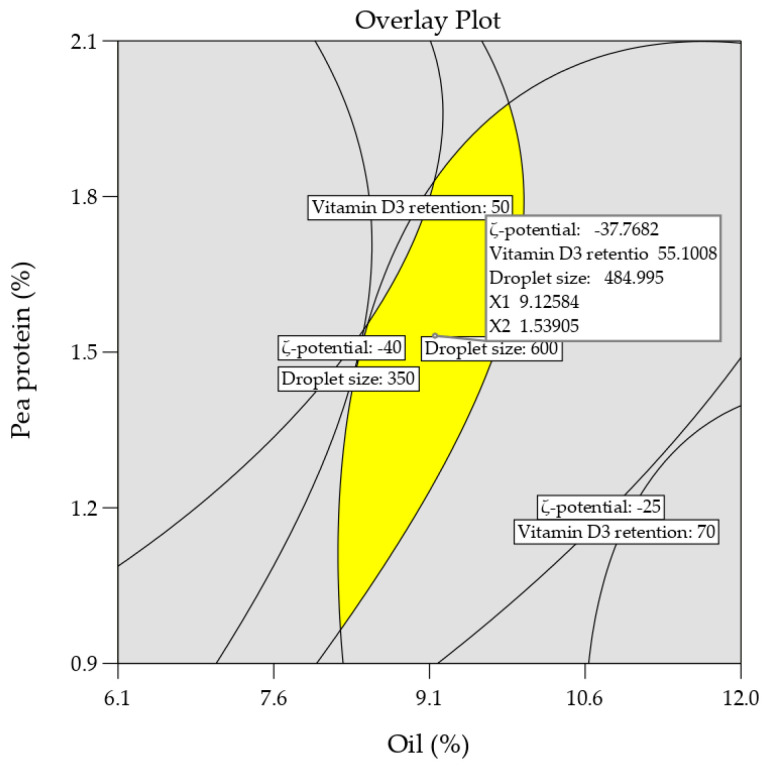
The overlay plot of the optimized nanoemulsion containing pea protein, safflower oil, and salt.

After developing the regression equations and studying the effects and interactions of the components of the nanoemulsions (SO, PP, and salt) on the ζ-potential, particle size, and retention of vitamin D3, the next step was to optimize the factors that lead to a more stable emulsion with a greater capacity for retaining vitamin D3. Applying RSM, the graphical optimization procedures were carried out to predict the optimal combination of SO, PP, and salt needed to prepare a nanoemulsion with a minimum value of ζ-potential, particle size, and maximum value of vitamin D3 retention. Contour plots for each response variable were superimposed to obtain an overlay plot. A region that satisfied the limits proposed was selected as the optimal combination of the independent factors studied. The resulting overlay plot is shown in Figure 2.

According to the results, the optimal combination of the independent factors was as follows: safflower oil = 9.12%, pea protein = 1.54%, and salt content = 0.4%. The predicted values of the ζ-potential, particle size, and vitamin D3 retention were −37.76 mV, 485 nm, and 55.1%, respectively. 

The optimal conditions for preparing the nanoemulsion were experimentally tested in triplicate, resulting in values of −36.5 ± 1.91 mV, 468 ± 17 nm, and 57.0 ± 2.54% for the ζ-potential, particle size, and vitamin D3 retention, respectively. The predicted and experimental values were similar (*p* > 0.05) when compared using a *t*-test. The optimized nanoemulsion obtained in this study can fortify certain foods, such as meat [24]. The following section discusses the findings regarding how vitamin D3 retention is affected when incorporated into a meat batter with varying percentages of safflower oil after cooking at 71 °C. It also focuses on the significance of encapsulating the vitamin D3.

### 3.3. Stability of Vitamin D3 in a Meat Batter after Cooking

This part of the study evaluated the effect of the vitamin D3 condition (encapsulated in a nanoemulsion or unencapsulated) in a functional meat model system on the vitamin D3 retention after cooking. The ζ-potential, particle size, and vitamin D3 retention in the nanoemulsion incorporated into the functional meat model system were −36.5 ± 1.91 mV, 468 ± 17 nm, and 57.0 ± 2.54%, respectively. These values were obtained from the optimization process in Section 3.2.

**Figure 3:**
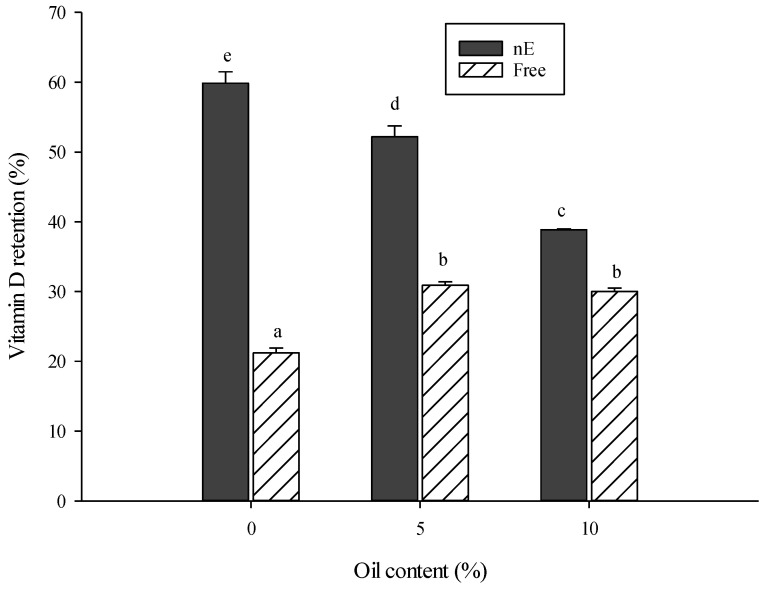
Vitamin D3 retention (%) in a meat batter with different fat contents. Vitamin D3 was incorporated encapsulated in a nanoemulsion (nE) or unencapsulated (free). Different letters denote significant differences (*p* < 0.05).

Figure 3 shows the results of vitamin D3 retention in a meat batter affected by the safflower oil content and the vitamin D3 incorporation condition. The effects of the interaction between the type of vitamin D3 incorporation (encapsulated or unencapsulated) and the percentage of SO (0, 5, 10%) in the meat batter were observed (*p* < 0.05). The treatments with the nanoencapsulated vitamin D3 had a higher recovery percentage (59.84–38.88%) compared to the unencapsulated form (30.88–21.21%) at all levels of SO incorporation (*p* < 0.05). This difference could be due to the smaller particle sizes in the nanoemulsions, which increases the surface area of the oil globules carrying the micronutrient. As a result, greater coating of the oil droplets with the surfactant is possible, leading to the improved stability and recovery of vitamin D3 in the system [28]. Similar results were found in a study by Syama et al. [39] on the fortification of milk with vitamin D2, either freely incorporated or complexed with caseins. After sterilizing the product, the authors reported a higher recovery of the complexed vitamin D2 (79.62−80.06%) than the free form (67.29−71.38%). Keršienė et al. [40] designed a yogurt enriched with vitamin D, incorporated freely or in a double emulsion (W/O/W). They reported a much higher recovery in treatments with the encapsulated vitamin D (94.04%) compared to the unencapsulated form (50.32%). Tippetts et al. [41] observed a recovery of emulsified vitamin D3 of up to 78% in a fortified cheese, while when incorporating the vitamin in free form, only 58% recovery was achieved. Therefore, nanoencapsulation is a viable and effective strategy to protect vitamin D3 from thermal degradation while processing food products, including meat products.

On the other hand, in Figure 3, it can also be seen that the recovery of nanoencapsulated vitamin D3 decreases by up to 21% as the incorporation of SO increases (*p* < 0.05). This could be because, with an increasing SO incorporation, the system has a lower amount of protein. This could have caused a more significant aggregation of the oil droplets, so the surface area available to be coated by the surfactant would be less, affecting the system’s stability and leading to a more extensive vitamin D3 degradation during processing. Walia and Chen [31] reported that a higher amount of oil and a lower amount of protein caused larger particle sizes, affecting the stability of the nanoemulsions loaded with vitamin D3. 

It is possible that other factors are involved in the stability and recovery of vitamin D3 in meat systems. An increase in SO results in a smaller amount of protein in the meat matrix, leading to weaker networks [42]. A weaker structure may allow greater oxygen access, promoting higher oxidation and a lower recovery of vitamin D3 [43]. Additionally, the heme iron present in the meat could accelerate oxidative reactions in the systems with a higher amount of SO [44]. It is also likely that the pH of the media played a role in the decrease in vitamin D3 recovery; the pH of our meat model system was around 6.0, and it has been reported that a pH ≤ 6 reduces the stability of the vitamin D3 complexes made of lipids and proteins, thereby reducing the system’s ability to protect the vitamin D3 from degradation [45].

To our knowledge, this is the first study that explores the incorporation of nanoencapsulated vitamin D3 into a meat system. It is essential to highlight that the present study only evaluates the influence of the incorporation method of the vitamin D3 and the SO percentage, and the results of the vitamin D3 retention were only explained in terms of these factors. However, other factors could impact the vitamin D3 stability. Although it is important to evaluate the influence of the main components involved in the functionality of food systems (such as proteins and lipids), it is well known that light, interaction with oxygen, an acidic medium, and the oxidation of lipids and proteins, could affect the vitamin D3 stability and retention during food processing, depending on the food matrix [46,47,48]. Meat matrixes are complex systems formed by several components such as water, proteins, lipids, minerals, and vitamins [49]. Processed meat products also contain other ingredients like salt and nitrites [50]. Moreover, meat and meat products are highly susceptible to oxidization because heme iron can catalyze lipid oxidation [44]. Hence, to elucidate the process of stability and retention of vitamin D3 in meat systems, it is recommended that the influence of the aforementioned factors be investigated in future analyses.

## 4. Conclusions

In this study, we optimized the composition of a nanoemulsion carrying vitamin D3 by using natural food components that could be used in functional meat products. The optimal condition was obtained by combining 1.54% pea protein, 9.12% safflower oil, and 0.4% salt, which resulted in a nanoemulsion with a ζ-potential of −36.5 ± 1.91, a droplet size of 468 ± 17 nm, and a vitamin D3 retention of 57.0 ± 2.54%. The vitamin D3, both encapsulated in the optimized nanoemulsion and unencapsulated, were incorporated into a meat batter formulated with different levels of safflower oil, and the vitamin D3 retention was evaluated after cooking. The results showed that incorporating nanoencapsulated vitamin D3 ensured a higher stability after cooking than the unencapsulated form. However, the meat batter formulated with safflower oil resulted in reduced vitamin D3 stability. The findings of this research provide valuable insights for developing new functional meat products using encapsulated VD3 with natural food components. The information generated in this study represents a starting point in developing innovative meat product addition and fortification strategies, contributing to increasing the variety and availability of foods enriched with this vitamin. Further research is needed to improve our understanding of incorporating vitamin D3 into meat products. This involves studying the effects of various factors, such as lipid oxidation, on the stability and preservation of vitamin D3, as well as the effects on meat product quality. Other areas of study should include the impact of processing methods, storage stability, bio-accessibility, and bioavailability.

## Figures and Tables

**Table 1:** Coded and experimental combinations of the treatments used for the central composite design and the experimental response variables measured in the nanoemulsions.

	Coded Values			Experimental Values			Response Variables		
Run	A	B	C	^a^ A: Oil	^b^ B: Protein	C: Salt	ζ-Potential	Particle Size	^c^ VD3
				%	%	%	(mV)	(nm)	Retention (%)
1	−1	−1	−1	7.03	0.91	0.10	−42.96	542	60.4
2	+1	−1	−1	12.97	0.91	0.10	−10.97	934	35.5
3	−1	+1	−1	7.03	2.09	0.10	−33.12	559	60.2
4	+1	+1	−1	12.97	2.09	0.10	−9.67	494	38.0
5	−1	−1	+1	7.03	0.91	0.40	−38.91	282	25.1
6	+1	−1	+1	12.97	0.91	0.40	−4.85	1830	79.9
7	−1	+1	+1	7.03	2.09	0.40	−43.86	215	35.1
8	+1	+1	+1	12.97	2.09	0.40	−28.59	1256	38.2
9	−1.68	0	0	5	1.5	0.25	−42.88	287	26.9
10	+1.68	0	0	15	1.5	0.25	−5.07	511	42.8
11	0	−1.68	0	10	0.5	0.25	−8.80	1631	58.4
12	0	+1.68	0	10	2.5	0.25	−26.21	791	36.2
13	0	0	−1.68	10	1.5	0.00	−27.48	603	38.5
14	0	0	+1.68	10	1.5	0.50	−29.92	355	68.5
15	0	0	0	10	1.5	0.25	−30.65	940	78.2
16	0	0	0	10	1.5	0.25	−34.06	560	60.3
17	0	0	0	10	1.5	0.25	−35.28	450	59.7
18	0	0	0	10	1.5	0.25	−32.50	961	57.2
19	0	0	0	10	1.5	0.25	−26.56	644	50.4
20	0	0	0	10	1.5	0.25	−41.02	433	58.3

Treatments 1–8 are factorial points, 9–14 are axial points, 15–20 are central points. ^a^ Safflower oil, ^b^ pea protein, ^c^ vitamin D3 retention.

**Table 2:** Estimated regression coefficient (RC), and analysis of variance of the regression models for the response variables measured in the nanoemulsions.

	ζ-Potential		Particle Size		VD3 Retention	
	RC	SE	RC	SE	RC	SE
Intercept	−33.22	1.95	663.1	112.9	60.62	4.61
A: ^a^ SO	12.33 *	1.29	241.1 *	74.9	2.76	3.06
B: ^b^ PP	−3.43 *	1.29	−181.5 *	74.9	−4.89	3.06
C: Salt	−1.73	1.29	46.6	74.9	2.52	3.06
AB	−3.42	1.69	−120.5	97.9	−6.13	4.00
AC	−0.76	1.69	282.8 *	97.9	13.13 *	4.00
BC	−4.98 *	1.69	−27.1	97.9	−4.26	4.00
A^2^	2.50	1.26	−80.1	72.9	−8.65 *	2.98
B^2^	4.79 *	1.26	206.8 *	72.9	−4.27	2.98
C^2^	0.83	1.26	−51.9	72.9	−2.06	2.98
R^2^	0.93		0.79		0.74	
*p*-values						
Regression	<0.001		0.018		0.04	
Lack of fit	0.53		0.27		0.24	

^a^ Safflower oil, ^b^ pea protein, * *p* < 0.05.

## Data Availability

The original contributions presented in the study are included in the article, further inquiries can be directed to the corresponding author.
